# Cryo-electron microscopy of extracellular vesicles in fresh plasma

**DOI:** 10.3402/jev.v2i0.21494

**Published:** 2013-12-31

**Authors:** Yuana Yuana, Roman I. Koning, Maxim E. Kuil, Patrick C. N. Rensen, Abraham J. Koster, Rogier M Bertina, Susanne Osanto

**Affiliations:** 1 Department of Clinical Oncology, Leiden University Medical Center, Leiden, The Netherlands; 2 Section of Electron Microscopy, Department of Molecular Cell Biology, Leiden University Medical Center, Leiden, The Netherlands; 3 Leiden Institute of Chemistry, Leiden University, Leiden, The Netherlands; 4 Department of Endocrinology and Metabolic Diseases, Leiden University Medical Center, Leiden, The Netherlands; 5 Einthoven Laboratory for Experimental Vascular Medicine, Leiden University Medical Center, Leiden, The Netherlands

**Keywords:** lipid vesicle, lipid bilayer, lipoprotein, microvesicle, exosome, platelet

## Abstract

**Introduction:**

Extracellular vesicles (EV) are phospholipid bilayer-enclosed vesicles recognized as new mediators in intercellular communication and potential biomarkers of disease. They are found in many body fluids and mainly studied in fractions isolated from blood plasma in view of their potential in medicine. Due to the limitations of available analytical methods, morphological information on EV in fresh plasma is still rather limited.

**Objectives:**

To image EV and determine the morphology, structure and size distribution in fresh plasma by cryo-electron microscopy (cryo-EM).

**Methods:**

Fresh citrate- and ethylenediaminetetraacetic acid (EDTA)-anticoagulated plasma or EV isolated from these plasmas were rapidly cryo-immobilized by vitrification and visualized by cryo-EM.

**Results:**

EV isolated from fresh plasma were highly heterogeneous in morphology and size and mostly contain a discernible lipid bilayer (lipid vesicles). In fresh plasma there were 2 types of particles with a median diameter of 30 nm (25–260 nm). The majority of these particles are electron dense particles which most likely represent lipoproteins. The minority are lipid vesicles, either electron dense or electron lucent, which most likely represent EV. Lipid vesicles were occasionally observed in close proximity of platelets in citrate and EDTA-anticoagulated platelet-rich plasma. Cryo-electron tomography (cryo-ET) was employed to determine the 3D structure of platelet secretory granules.

**Conclusions:**

Cryo-EM is a powerful technique that enables the characterization of EV in fresh plasma revealing structural details and considerable morphological heterogeneity. Only a small proportion of the submicron structures in fresh plasma are lipid vesicles representing EV.

Extracellular vesicles (EV) are a heterogeneous population of vesicles surrounded by a phospholipid bilayer with diameters less than 1 µm (1). They include microvesicles (microparticles), exosomes, and apoptotic vesicles (1). They are released from a variety of cells via pathways involved in cellular activation, stress and apoptosis. Long considered as inert cellular debris, EV are increasingly recognized as important mediators of cellular cross-talk in various biological fluids including blood, urine and synovial fluid. EV are present in the plasma of healthy subjects and at elevated concentrations in plasma of individuals with various diseases (2,3).

To date, there is only limited information on the morphology, composition and size distribution of EV in fresh plasma because of limitations of available analytical methods (4), whereas there is limited information on the effects of pre-analytical variables such as blood collection, plasma and EV preparation and storage (5–7). Flow cytometry is still the most commonly applied method to study EV in citrated plasma or in fractions isolated from citrated plasma by multiple rounds of centrifugation. The disadvantage of flow cytometry is the limitation of the wavelength of the laser light to accurately and reliable study vesicles sized less than 0.5 µm (4,8,9).

Recently, the application of novel analytical methods like atomic force microscopy (AFM) and nanoparticle tracking analysis indicated that the large majority of plasma EV has a size much smaller than previously reported using flow-cytometry (8,9). These novel methods can sensitively detect EV, in particular those with a diameter smaller than 0.5 µm. However, they provide no information about the morphology and structure of EV.

Conventional transmission electron microscopy (TEM) has been used to study the morphology of EV isolated from plasma (10,11), significant shortcomings being that the sample preparation steps and imaging techniques require dehydration, chemical fixation and/or staining of the biological specimens. To study the morphology of EV in fresh plasma cryo-electron microscopy (cryo-EM) seems more suitable than conventional TEM. Cryo-EM does not use staining or chemical fixation procedures and samples are directly applied onto an EM grid, vitrified and visualized. Vitrification is a cryo-fixation method to preserve biological specimens to near-atomic resolution (12) while water is transformed into a glass-like state without the formation of ice crystals. Thus, cryo-EM of vitrified biological specimens, small molecular fragments or whole cells enables observation of biological structures in a vitrified near-native state (13,14). Cryo-EM also allows 3D tomographic data collection thus enabling the spatial visualization of more complex structures.

Using cryo-EM, we studied the size and morphology of EV as they occur in fresh human plasma. The majority of studies reported on EV in human blood have been performed using citrated blood (5). We used both fresh citrated blood and EDTA blood to prepare plasma and to isolate EV. EDTA is a much stronger chelator of Ca^2 +^ and Mg^2+^ ions than citrate, which effectively prevents biochemical and cellular activation reactions.

In this study, we demonstrated that cryo-EM enables the characterization of individual EV in their native state in fresh human plasma. Our data indicate that in fresh plasma most particles are lipoproteins, while only a small fraction could be identified as EV.

## Materials and methods

### Blood collection

After fasting for more than 12 hours, venous blood was collected from 2 healthy volunteers (1 female and 1 male, 34 and 57 years old, respectively), who gave informed consent to donate blood. Blood was drawn using a 21-G needle. The first 5 mL of blood were discarded. The next 4.5 mL were collected into a 5-mL sodium citrate (0.105 M) vacutainer tube, after which the next 10 mL were collected into K_2_EDTA (1.8 mg EDTA/mL blood). Needles and blood collection tubes were purchased from BD Biosciences, USA. Blood was further processed immediately, within 15 minutes after collection of blood in citrate or EDTA, preventing clotting of blood and cellular activation (in case of EDTA).

### Plasma preparation

We prepared platelet-rich, platelet-poor and platelet-free plasma (PRP, PPP, PFP, respectively) from citrate- and EDTA-anticoagulated plasma to either rapidly cryo-immobilize plasma by vitrification or isolate EV by high-speed centrifugation. Blood was centrifuged at 160 g, 20°C for 13 minutes without brake to prepare PRP. For PPP preparation, blood was centrifuged twice at 2,000 g, 20°C for 10 minutes without brake. PFP was prepared by centrifuging the blood first at 1,500 g, 20°C for 15 minutes and then at 13,000 g, 20°C for 2 minutes, without brake. During aspiration of supernatant plasma, about 700 µL plasma was left above the white blood cell layer to avoid contamination of the plasma with white cells and platelets.

### Isolation of EV from plasma

EV were isolated from 750 µL of fresh citrated or EDTA PPP by centrifugation at 18,890 g, 20°C for 30 minutes (8). The supernatant (725 µL) was carefully removed without disturbing the pellet. Next, this pellet was resuspended in 1 mL Hepes buffer (10 mM Hepes, 137 mM NaCl, 4 mM KCl (all from Merck, Germany), 0.1 mM Pefabloc SC (Fluka, Germany), pH 7.4) and centrifuged again at 18,890 g, 20°C for 30 minutes. Finally, the supernatant was removed leaving an undisturbed pellet in 25 µL of buffer. The pellet consisting of isolated EV was carefully resuspended and processed for cryo-EM by rapid vitrification. The EV concentration in the suspension was 30-fold more concentrated than the original plasma EV, while the suspension of isolated EV contained 40-times less plasma proteins.

### Isolation of lipoproteins from plasma

Very low density lipoproteins (VLDL; d<1.006 g/mL), low density lipoproteins (LDL; 1.019<d<1.063 g/mL), high density lipoproteins (HDL; 1.063<d<1.21 g/mL) and lipoprotein-deficient plasma (LPDP; d>1.21 g/mL) were isolated from 3.5 mL EDTA PFP collected from a fasted healthy volunteer by density-gradient ultracentrifugation according to Redgrave et al. (15). Commercially available VLDL, LDL and HDL isolated from human plasma were purchased from Merck Millipore, Germany.

### Cryo-electron microscopy

Citrate- or EDTA-plasma samples for cryo-EM were immediately processed after blood collection: all plasma samples were put on the grid and vitrified within 15 minutes after plasma preparation (i.e. within 2 hours after citrate- or EDTA-anticoagulated blood collection). Samples were applied on a glow-discharged (2 minutes in 0.2 mbar air using a EMITECH K950X with glow discharger unit) 300 mesh EM grid with lacey carbon (01883-F, Ted Pella) and were vitrified using a Vitrobot Mark IV (FEI Company, The Netherlands) or a Leica EM GP (Leica, Germany) at room temperature and 100% humidity. Excess sample was removed by blotting once between 1 and 2 seconds with filter paper. The blotted grids were plunged into liquid ethane that was kept in equilibrium with solid ethane. After vitrification the grid was stored under liquid nitrogen until further use. Grids were mounted in a Gatan 626 cryo-holder for cryo-EM imaging.

Two dimensional automated data collection for quantification (Table I) was performed on a Tecnai 12 electron microscope (FEI Company, The Netherlands) operated at 120 kV. Images were recorded on a 4 k×4 k Eagle camera (FEI Company, The Netherlands). Images were recorded at 13,000× magnification (pixel size 2.3 nm) at 8 µm under focus. For large-scale automatic data collection we used myTEM software (16). In each experiment, 1,000 non-overlapping images (in total ~3,000 µm^2^) were recorded. Images were processed by using IMOD (the Boulder Laboratory for 3-D EM of Cells, Colorado, USA), a set of image processing, modelling and display programs used for tomographic reconstruction and for 3D reconstruction of EM serial sections and optical sections. All particles with a diameter equal or above 25 nm in EDTA plasma (PRP, PPP and PFP) were counted from ~3,000 µm^2^ images and the size distribution determined (Table II).

**Table I:** PRP, PPP and PFP were freshly isolated from EDTA plasma of 2 fasted healthy volunteers (1: female; 2: male) and processed immediately for cryo-EM imaging. Quantification of all particles, lipid vesicles and platelets.

		Counts per ~3,000 µm^2^		
		
Volunteer	EDTA plasma	All particles	Lipid vesicles	Platelets
1	PRP	620	35	14
	PPP	7,580	2	0
	PFP	1,320	10	0
2	PRP	2,210	13	41
	PPP	3,610	3	0
	PFP	6,590	5	0

Cryo-electron tomography and 2D imaging was performed on a Tecnai 20 FEG (FEI Company, The Netherlands) operated at 200 kV. Images were recorded on a 2 k×2 k camera mounted behind a GIF energy filter operated at a slit width of 20 eV. Images were recorded at a magnification between 19.000× (1.6 nm pixel size) and 95.000× (0.32 nm pixel size). Cryo-electron tomograms were recorded from 60° at a defocus of -50 µm and a magnification of 3,600× corresponding with a pixel size of 3.55 nm.

## Results

### EV in plasma

**Fig. 1:**
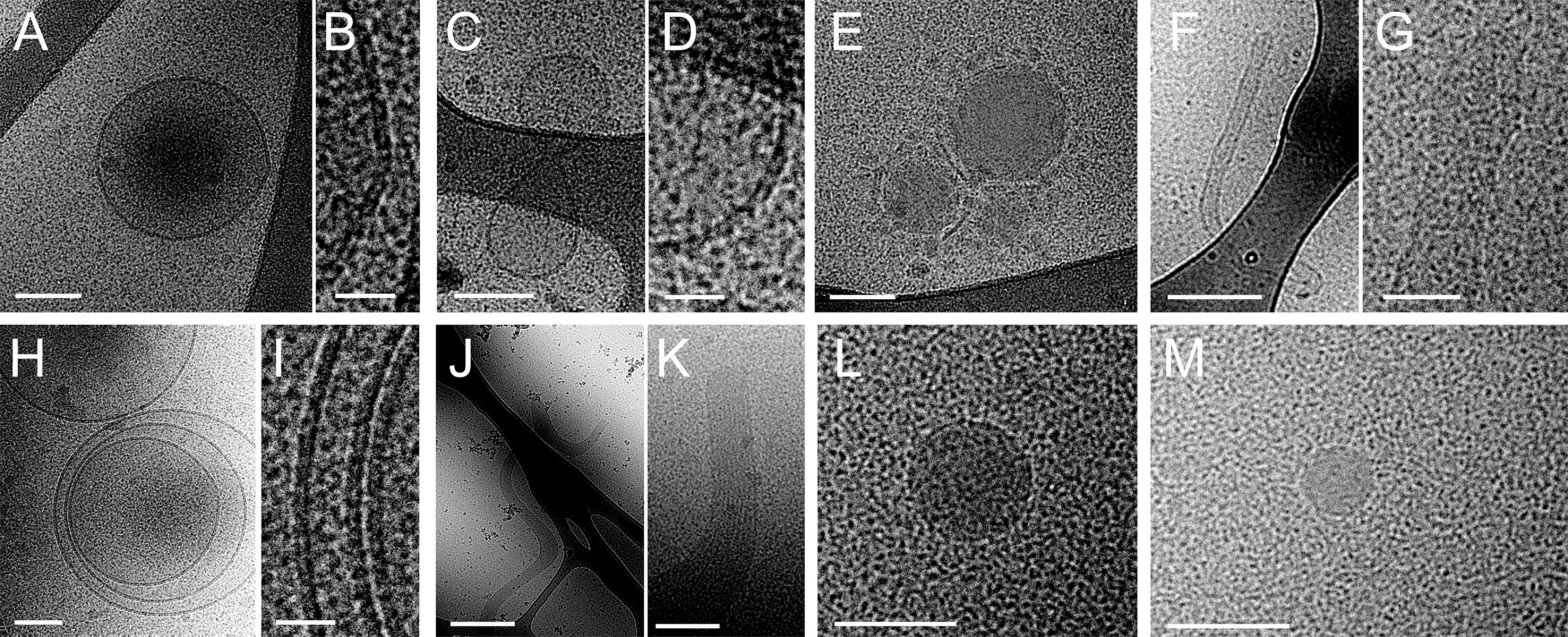
Cryo-EM images of EV in platelet-rich plasma. Differently shaped particles were observed by cryo-EM: round (A) or oddly shaped (C) empty particles with a clear lipid bilayer (resp. B, D) and electron dense vesicles surrounded by smaller spherical structures (E). Also, large elongated empty vesicles (F, G), multilayered vesicles (H, I) and elongated (presumably actin) filled vesicles (J, K) were observed. Electron dense particles, which are likely lipoprotein particles, are observed frequently (L, M). Scale bars are 25 nm (B, D, I), 50 nm (G), 100 nm (A, C, E, H, K, L, M), 250 nm (F) and 500 nm (J).

**Fig. 2:**
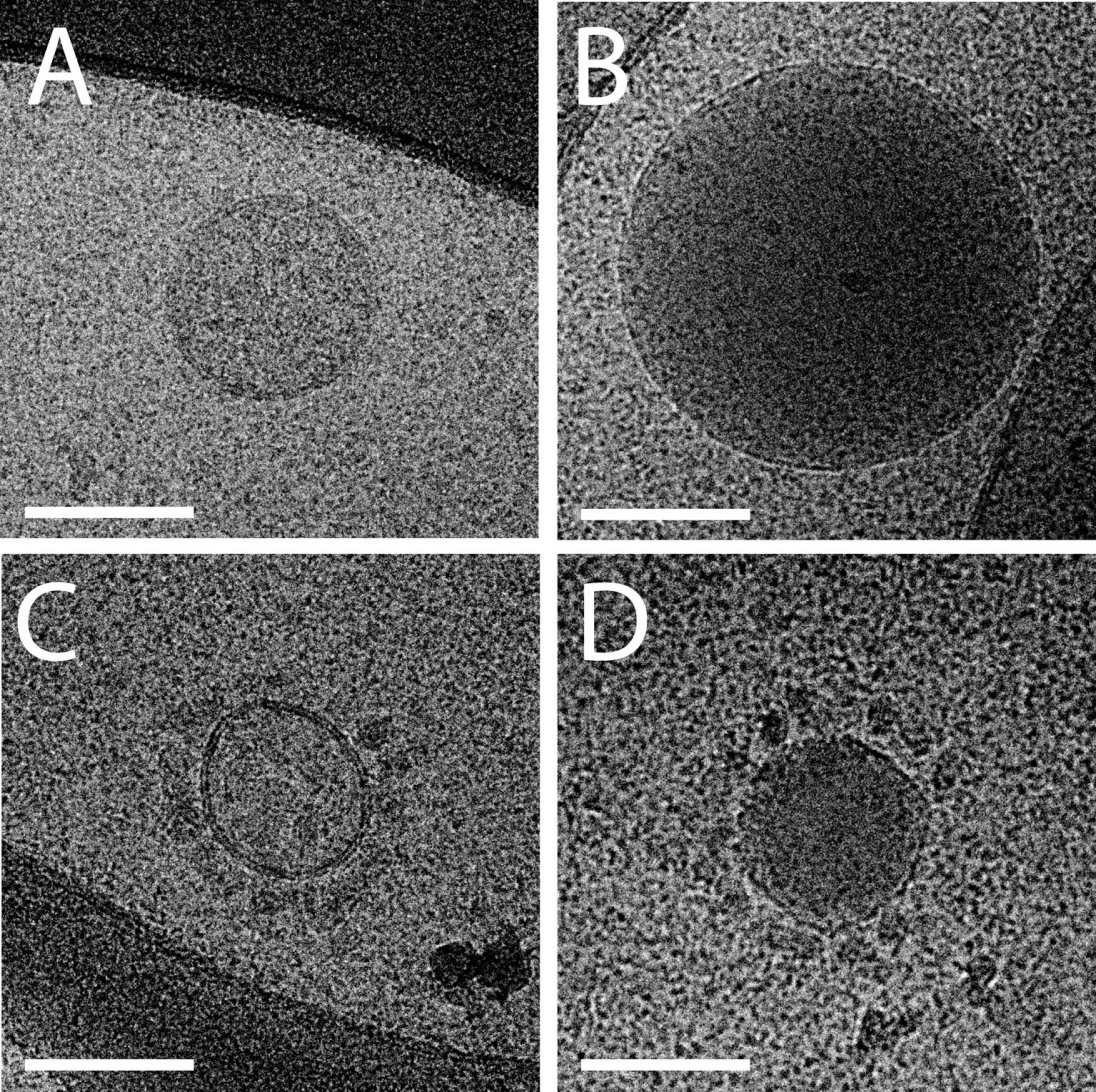
Cryo-EM images of EV in platelet-poor plasma. In PPP, some large electron lucent vesicles with a clearly discernible lipid bilayer are present (A and C). Other particles are electron dense with a clear lipid bilayer (B and D). Some vesicles are surrounded by smaller spherical structures (C and D). Scale bars are 100 nm.

In citrated and EDTA plasma (PRP, PPP and PFP), EV were more uniform in type and less variable in shape. We found 3 distinct dominant structural types. Some of the particles in PRP had a clear lipid bilayer/membrane and were typically translucent with little material inside (further referred to as “lipid vesicles,” see Fig. 1A–E). Most particles had a round shape with an electron dense structure without an apparent lipid membrane (Fig. 1L and M). Vesicles surrounded by smaller spherical structures (Fig. 1E), multilayer vesicles (Fig. 1H and I) and elongated filled vesicles (Fig. 1J and K) were also found. The same types of structures were observed in PRP, PPP and PFP. Noticeably, PPP and PFP seem to contain a less heterogeneous population of particles than PRP (Fig. 2). It should be noted that plasma samples have a high protein concentration, which is reflected in all cryo-EM images by a grainy background appearance.

**Fig. 3:**
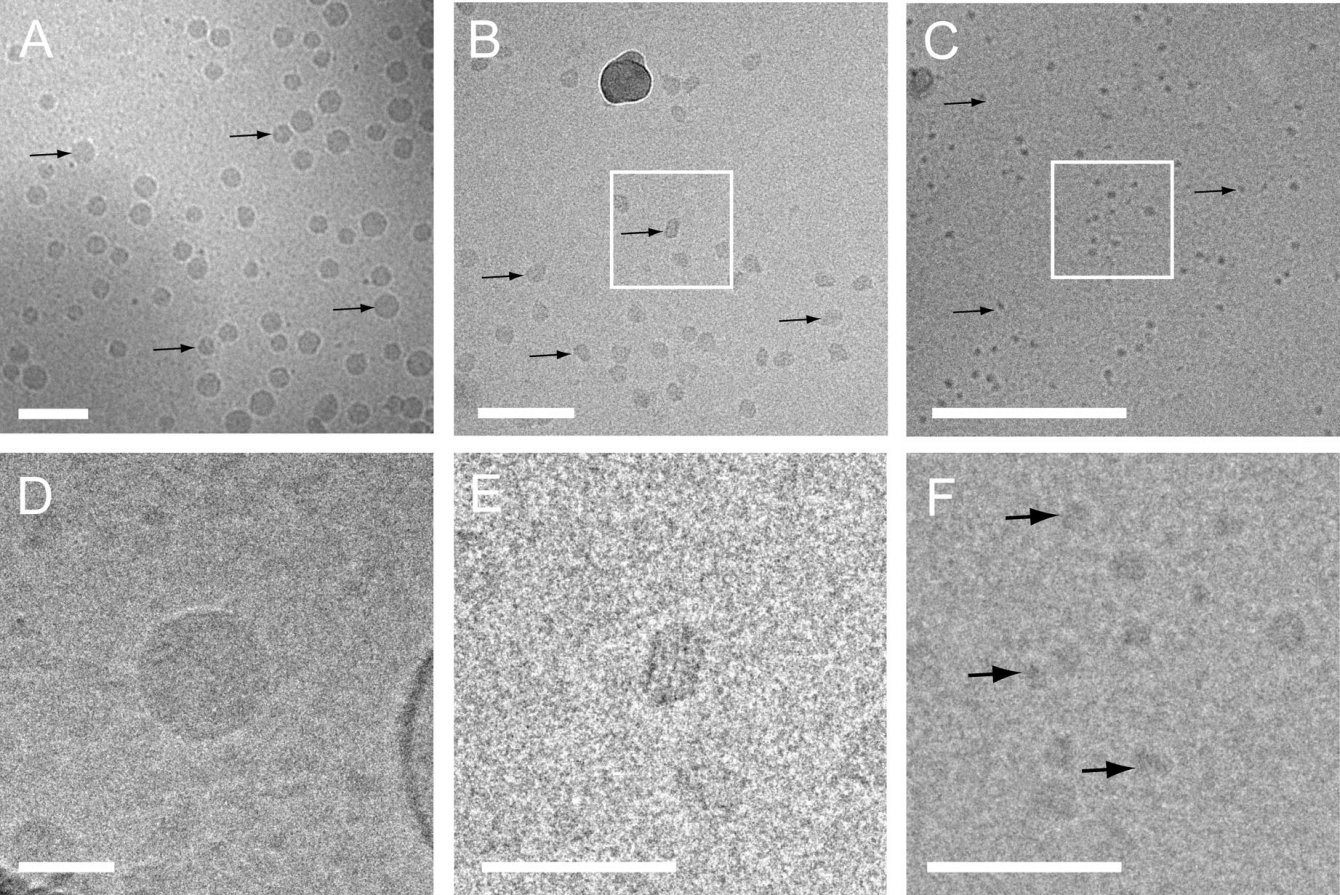
Cryo-EM of freshly isolated lipoprotein fractions. Overviews (A–C) and higher magnification (D–F) images of VLDL (A and D), LDL (B and E) and HDL (C and F) fractions clearly show the different lipoprotein particles (denoted by black arrows). White squares in B and C denote magnified images in E and F. Also note the periodic layers of LDL in E. Scale bars are 100 nm (A and B) and 50 nm (C, D, E and F).

In general, most particles visualized in plasma were round, electron dense and without a visible lipid bilayer (Fig. 1L and M). The absence of a visible lipid bilayer, the high abundance of these electron dense particles and their wide range in size distribution (from ~25 nm to a few hundred nanometres), strongly suggested that these particles represent lipoproteins such as VLDL (30–90 nm) and LDL (~25 nm). To test this, the structure of these particles was compared with those of lipoprotein fractions that were isolated from EDTA PFP by density-gradient ultracentrifugation and imaged using cryo-EM (Fig. 3). Cryo-EM imaging of these lipoprotein fractions and also earlier published cryo-EM data of lipoproteins (17–19) demonstrated that the electron dense particles without apparent lipid bilayer observed in plasma samples and to a lesser extent in isolated EV resembled lipoproteins in size and morphology. Moreover, no particles were observed in the LPDP, confirming that these highly abundant electron-dense particles were indeed lipoproteins.

The majority of submicron particles found in EDTA plasma were electron dense and classified as lipoproteins, whereas a minor fraction (less than 1%) could be identified as lipid vesicles which most likely represent EV (Table I). We measured the diameter size of all particles in EDTA plasma and found that these particles have diameters ranging from 25 to 260 nm with a typical size of ~30 nm (Table II). This particle size confirms that lipoprotein particles are dominant in all plasma samples. In general, the particle size distribution was not largely influenced by the procedure used to prepare the plasma. However, particles in PRP seemed to cover a wider range of sizes.

**Table II:** PRP, PPP and PFP were freshly isolated from EDTA plasma of 2 fasted healthy volunteers (1: female; 2: male) and processed immediately for cryo-EM imaging. Size distribution of all particles. Particles with sizes above or equal to 25 nm in diameter were counted and measured to determine the size distributions. Platelets were not measured.

Volunteer	EDTA plasma	Median (nm)	Range (nm)
1	PRP	28	25–148
	PPP	28	25–75
	PFP	28	25–64
2	PRP	31	25–260
	PPP	32	25–68
	PFP	35	25–92

### EV isolated from plasma

Since most studies use EV isolated from citrate-anticoagulated blood, we also used EV isolated from fresh citrated blood for the cryo-EM studies. Like in plasma, we found a large variety of particles in these EV preparations that differed considerably in shape and electron density (Supplementary file S1). Similar observations were made for EVs isolated from EDTA-anticoagulated plasma. Most vesicles had a round shape with either electron lucent structure or a clear lipid bilayer (Supplementary file S1A) which were similar to the lipid vesicles observed in plasma (Fig. 1A and C) or with an electron dense structure without an apparent lipid membrane (Supplementary file S1B). Vesicles with various morphologies were also found and they were either electron lucent structures with a clear lipid bilayer (Supplementary file S1D, G, and I) or electron dense structures (Supplementary file S1C, E, and F). Surprisingly, actin filaments (Supplementary file S1H) and small microtubules (data not shown) were also observed, Most likely, citrate and EDTA do not completely prevent the activation of platelets during preparation of plasma which may result in the inadvertent release/formation of EV and other structures into the plasma.

### Platelets in fresh plasma

**Fig. 4:**
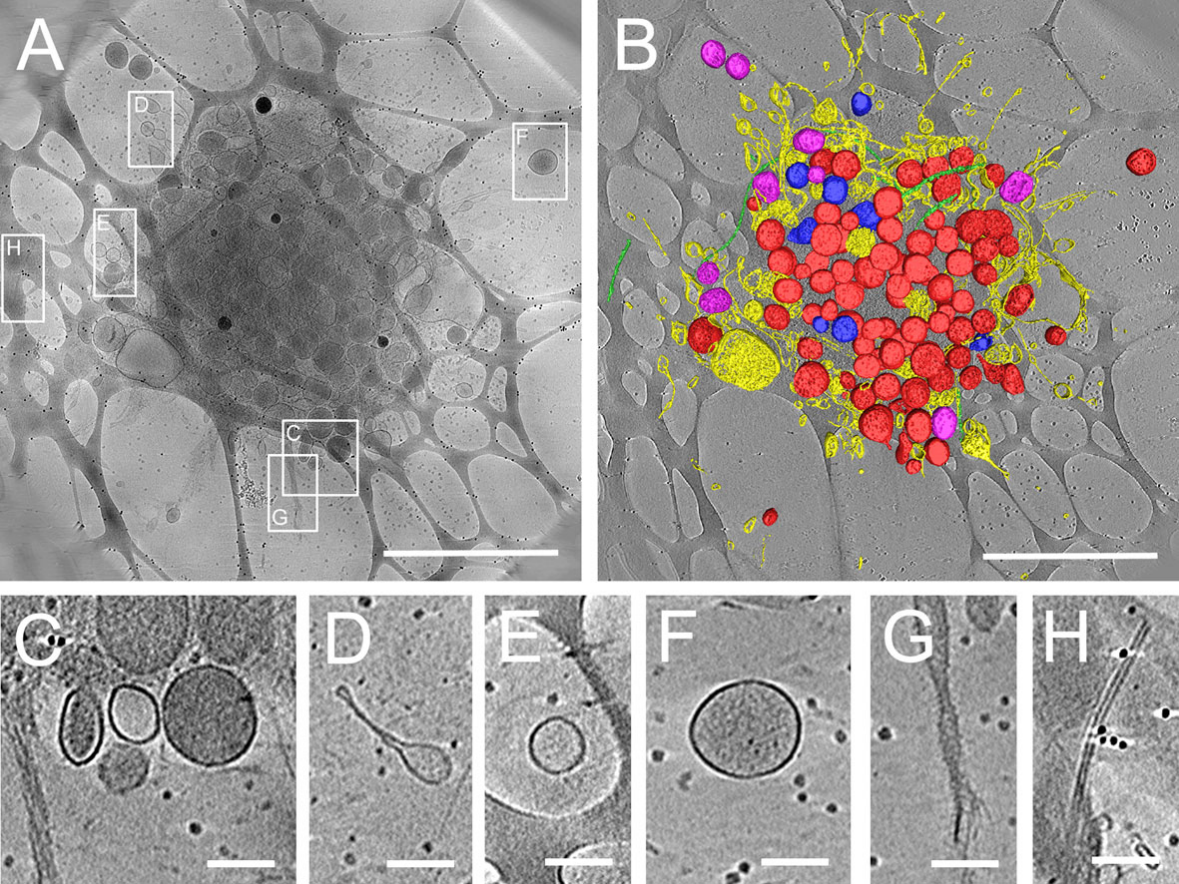
Three-dimensional tomogram of ruptured platelet and surrounding vesicles in EDTA plasma. 3D structure determination of ruptured platelets in cryo-EM was obtained by tomographic data collection thus bypassing potential inaccuracies of 2D projection images of thick platelets. Cryo-electron tomography shows several types of vesicular structures and cellular debris that can be found in the vicinity of ruptured platelets. In (A), a 175 nm thick tomographic slice of a ruptured platelet showing the debris that is released. Boxed areas show the positions of the enlarged images C–H. A surface rendered representation (B) shows: dense granules (blue), which have highly dense inclusions inside; vesicles and lipid membrane structures (yellow); alpha-granules (red), which are more electron dense than other vesicles; mitochondria (purple) and microtubules (green). Small black dots in the background are fiducial gold used for tilt series alignment. The network-like structure in the background is the lacey carbon film that is used as support film. Enlargements of several places surrounding the activated platelet show release of dense vesicles (C and F), round vesicles (C, E and F) and elongated lipid membrane structures (D), rough membranous structures (G) and microtubules (H). Scale bars in A and B are 2 µm, in C–H scale bars are 200 nm.

In addition, we performed cryo-EM tomography to visualize the structure of lipid vesicles in proximity of platelets. Fig. 4 shows a 3D image of the structure of a platelet and the multitude of vesicles derived from platelets obtained by cryo-EM tomography of a platelet in EDTA PRP. Platelet organelles, like mitochondria, alpha-granules and dense granules, could be identified in the tomograms (Fig. 4B and C). These structures were morphologically similar to those reported in earlier morphological studies of platelets (20). Interestingly, some vesicles were found attached to or near the platelet outer membrane (Fig. 4B, D, E and G). Vesicles were also present in the vicinity of a platelet (Fig. 4B, C and F), while microtubules (Fig. 4B and H) were visible in- and outside the platelet. A movie of the cryo-EM reconstruction of the 3D structure of a platelet is provided as a Supplementary movie M1.

## Discussion

In this study, we demonstrate that cryo-EM enables visualization and characterization of the morphology of EV both in fractions that are isolated from human plasma as well as in fresh human plasma. We studied plasma isolated from citrate and EDTA-anticoagulated blood using commonly used operational procedures. In plasma, there are mainly electron dense lipoprotein particles, whereas only a small portion consists of lipid vesicles. All particles in plasma have diameters ranging from 25 to ~300 nm. Smaller particles below 25 nm which can be protein complexes and other lipoproteins (i.e. HDL; 8–10 nm) were also present, but more difficult to detect unambiguously because of limited contrast due to the technique of cryo-EM and the relatively large amount of proteins present in plasma.

Using conventional EM, Aras et al. (10) observed that vesicles isolated from plasma had diameter sizes ranging from 100 to 500 nm. Using cryo-EM, we generally found smaller vesicles in plasma samples but also some within the size range reported by Aras et al. The small difference in observed size range might originate from the difference in sample preparation steps. In conventional EM, the sample is dehydrated before imaging. During drying, EVs may collapse, resulting in a cup-shaped morphology (3). In cryo-EM, the sample remains fully hydrated and is quickly vitrified and as a result, EVs have a round shape (21). In cryo-EM experiments, flattening of soft particles can occur when particles are larger than the specimen thickness, which is typically around 200–300 nm.

In plasma, only 2 types of particles were observed: lipoprotein particles and lipid vesicles. The lipid vesicles have a similar morphology than the lipid vesicles present in isolated EV (Supplement file S1). Additionally, some lipid vesicles in plasma (Fig. 1A and 2A, B) had similar morphology than some vesicles found in the vicinity of platelets (Fig. 4C–F), suggesting that they might originate from platelets. The use of specific antibody-based labelling techniques, which is beyond the scope of the present study, will be useful to quantify the subset of populations among these lipid vesicles (22).

We are aware that the procedures used for plasma preparation and for cryo-EM sample preparation, such as sample centrifugation and blotting, can cause (mechanical) activation of platelets. However, we did not see an increase in the numbers of lipid vesicles in PPP and PFP in comparison to PRP (Table I). Furthermore, for preparing our cryo-EM samples, we also have used a Leica EM GP, a vitrification system which allows blotting from one side, allowing more gentle blotting. Certainly, more experiments are necessary to investigate the effect of the anticoagulant on the vesiculation and morphological details of platelet activation using cryo-EM.

Cryo-EM provides improved specimen preservation and potentially more realistic morphology of biological structures. The trade-off for these improvements, compared to the more conventional techniques including (sectioning and) staining is the intrinsically higher sensitivity to electron beam radiation in cryo-EM, which results in a lower contrast, because it requires underexposure of photographs to prevent visible damage. Tomographic data collection provides not only accurate 3D reconstructions and localization of molecular and vesicular structures, bypassing the limitations and inaccuracies of projection imaging (Fig. 4), but also generates higher contrast by applying using more electron dose and generation of a 3D map.

We found particles with a diameter ranging from 25 to 260 nm (a median of about 30 nm) in the EDTA plasma samples of 2 healthy volunteers. Most of these particles were electron dense without a lipid bilayer, thus, resembling lipoprotein particles, whereas lipid vesicles formed the minority of the observed particles. Lipoprotein particles such as VLDL and LDL have reported sizes of ~25–90 nm, which might explain why the median size of particles in plasma observed by cryo-EM (30 nm) is lower than previously reported (8).

It should be noted that the specimen preparation method for cryo-EM, blotting and vitrification, might influence the number and size distribution of EV and lipoprotein particles in plasma samples. Larger or smaller particles might be preferentially extracted by the blotting paper leading to an underestimation of their quantity when analyzed by EM.

However, custom built software (16) enabled us to record a large number of particles at high resolution, enabling us to count particles with a size between 25 and 100 nm, providing some insight into the size distribution and morphology of EV in fresh plasma. We must also note that quantification of EV using cryo-EM depends on specimen preparation conditions. Local variations in concentrations of EV might occur due to, e.g. possible aggregation and preferential binding of EV to the EM support. Quantification with cryo-EM of EV might not be possible without prior concentration or purification of the sample.

Overall, our data indicate that EV constitute only a very small fraction of all submicron particles present in fresh plasma isolated from the blood of fasted healthy volunteers, whereas the majority are lipoprotein particles. Therefore, we recommend blood collection from fasting donors to limit the interference of lipoproteins, especially chylomicrons, with the quantification of EV directly in plasma as much as possible. In the future, cryo-EM may support and complement the measurement of EV by other technologies such as AFM using antibody-coated mica surfaces (8), nanoparticle tracking analysis (9) and/or scanning ion occlusion sensing (23). In particular, identification of subsets of EVs using antigen-specific gold-labelled monoclonal antibodies may be helpful to assess unambiguously the precise nature of EV and their cellular origin.
